# CLARITY and Light-Sheet microscopy sample preparation in application to human cerebral organoids

**DOI:** 10.18699/VJ21.103

**Published:** 2021-12

**Authors:** T.A. Shnaider, I.E. Pristyazhnyuk

**Affiliations:** Institute of Cytology and Genetics of the Siberian Branch of the Russian Academy of Sciences, Novosibirsk, Russia; Institute of Cytology and Genetics of the Siberian Branch of the Russian Academy of Sciences, Novosibirsk, Russia

**Keywords:** cerebral organoids, CLARITY, Light-Sheet microscopy, immunohistochemistry, tissue clearing, tissue imaging, церебральные органоиды, CLARITY, Light-Sheet микроскопия, иммуногистохимия, очистка тканей

## Abstract

Cerebral organoids are three-dimensional cell-culture systems that represent a unique experimental model reconstructing early events of human neurogenesis in vitro in health and various pathologies. The most commonly used approach to studying the morphological parameters of organoids is immunohistochemical analysis; therefore, the three-dimensional cytoarchitecture of organoids, such as neural networks or asymmetric internal organization, is difficult to reconstruct using routine approaches. Immunohistochemical analysis of biological objects is a universal method in biological research. One of the key stages of this method is the production of cryo- or paraffin serial sections of samples, which is a very laborious and time-consuming process. In addition, slices represent only a tiny part of the object under study; three-dimensional reconstruction from the obtained serial images is an extremely complex process and often requires expensive special programs for image processing. Unfortunately, staining and microscopic examination of samples are difficult due to their low permeability and a high level of autofluorescence. Tissue cleaning technologies combined with Light-Sheet microscopy allows these challenges to be overcome. CLARITY is one of the tissue preparation techniques that makes it possible to obtain opaque biological objects transparent while maintaining the integrity of their internal structures. This method is based on a special sample preparation, during which lipids are removed from cells and replaced with hydrogel compounds such as acrylamide, while proteins and nucleic acids remain intact. CLARITY provides researchers with a unique opportunity to study three-dimensional biological structures while preserving their internal organization, including whole animals or embryos, individual organs and artificially grown organoids, in particular cerebral organoids. This protocol summarizes an optimization of CLARITY conditions for human brain organoids and the preparation of Light-Sheet microscopy samples.

## Abstract (ru)

Церебральные органоиды – это трехмерные системы культивирования клеток, представляю- щие собой уникальную экспериментальную модель, которая позволяет реконструировать ранние события нейрогенеза человека in vitro в норме и при различных патологиях. На сегодняшний день для изучения морфологических параметров органоидов чаще всего применяют иммуногистохимический анализ. В связи с этим аспекты трехмерной цитоархитектуры органоидов, такие как нейронные сети или асимметричная внутренняя организация, трудно реконструировать при использовании рутинных подходов. Иммуногисто- химический анализ биологических объектов является универсальным методом в биологических исследова- ниях. Один из ключевых этапов данного подхода – изготовление крио- или парафиновых серийных срезов образцов. Это очень трудоемкий и времязатратный процесс. Кроме того, срезы представляют собой лишь небольшую часть исследуемого объекта, а трехмерная реконструкция из полученных серийных изображе- ний является крайне сложной процедурой и часто требует специальных дорогостоящих программ для об- работки изображений. К сожалению, окрашивание и микроскопирование целых образцов затруднено из-за их низкой проницаемости и высокого уровня автофлуоресценции. Технологии очистки тканей в сочетании с Light-Sheet микроскопией дают возможность преодолеть эти проблемы при работе. CLARITY – это одна из технологий подготовки тканей, позволяющая сделать непрозрачные биологические объекты прозрачными с сохранением целостности их внутренней структуры. Метод основан на специальной пробоподготовке, во время которой из клеток удаляются липиды и заменяются гидрогелевыми соединениями, такими как акриламид; при этом белки и нуклеиновые кислоты остаются интактными. Технология CLARITY предоставляет исследователям уникальную возможность изучать объемные биологические структуры с сохранением их внутренней организации, включая целых животных или эмбрионы, отдельные органы и искусственно выра- щенные органоиды, в частности церебральные. Данный протокол обобщает оптимизацию условий CLARITY для органоидов головного мозга человека и особенности подготовки образцов Light-Sheet микроскопии.

## Introduction

Biological tissues and organs present a complex three-dimensional structure. Due to their opacity and high level of autofluorescence, three-dimensional reconstruction of such objects is an extremely laborious, but necessary process. To date, a number of protocols (more than a dozen) have been developed for making tissue transparent: SeeDB (Ke et al., 2013), ScaleA2 (Hama et al., 2011), uDISCO (Pan et al., 2016), CLARITY (Chung, Deisseroth, 2013), CUBIC (Susaki et al., 2015) and others. In general, all protocols can be divided into three groups, depending on the chemicals used for tissue clearance: organic solvents (hydrophobic reagent)-based protocols (BABB, 3DISCO, ECi method), hydrophilic reagent-based protocols (ClearT, Scale, FUnGI, Fructoseglycerol, CUBIC and other) and hydrogel-tissue chemistry-based protocol (CLARITY, SWITCH and SHIELD) (Ueda et al., 2020; Susaki, Takasato, 2021). Some of them have different advantages like quality and speed of clearing or simplicity of the procedure. But on the other hand, some of the protocols involve using toxic and corrosive chemicals that require special objectives to avoid damage to the microscope or require other special equipment. Most of these protocols have been developed to clarify entire organs or their big fragments

Recently, a new method of artificial mini-organ or organoids generation from induced pluripotent stem cells (iPSC) was developed (Lancaster et al., 2013) and now many different types of organoids have already been produced (brain, lung, liver, intestine, pancreas, kidney and others). Organoids are widely used both to recreate the three-dimensional architecture and functional activity of the original organs during normal embryonic development and at various disorders and to test the biological activity of various drugs, chemical and biological agents. Usually, organoids are opaque, which makes investigating them rather difficult. For this purpose, it is advisable to use the combination of tissue clearing and 3D imaging technologies. However, it is important to select the clarifying technology that would match organoids size and fragility as much as possible and would produce sufficient resolution for investigation of tiny structures.

Various techniques have been used for organoid tissue clearing and several studies have compared different clarifying methods which could be applied to mini-organs (Susaki, Takasato, 2021). Some techniques, such as the hydrophilic clearing protocols (ClearT2 and ScaleS) are most acceptable for clearing small spheroids such as neurospheres (Boutin, Hoffman-Kim, 2015) or cancer cell spheroids (Boutin et al., 2018). Others, such as RapiClear, Fructoseglycerol and FUnGI, also using hydrophilic components, are designed and optimized for handling small and fragile, predominantly hollow organoid structures such as intestinal organoids. It should be noted that these protocols are very convenient and take only three days without application of harmful chemicals (Dekkers et al., 2019; van Ineveld et al., 2020). For complex and dense brain organoids, stronger clearing protocols including delipidation procedure are usually used (Susaki, Takasato, 2021). Applying organic solvent-based methods like 2Eci (2nd generation Ethyl cinnamate-based clearing method) (Masselink et al., 2019; Goranci-Buzhala et al., 2020) or BABB method for midbrain organoids (Renner H. et al., 2020) can get a relatively quick (within a few days) result. However, most of the organic components used in these protocols are quite toxic (for example, a mixture of benzyl alcohol and benzyl benzoate in BABB method (Renner H. et al., 2020)).

The use of hydrogel-tissue chemistry sometimes provides more opportunities for preserving the structure of organoids and increasing the optical resolution of tiny objects. That is due to the tissue hydrogel scaffold preparation by cross-linking hydrogel monomers to native biomolecules (Gradinaru et al., 2018). The creation of such a polymer frame in the brain organoids allows combining these protocols with additional procedures with sodium dodecyl sulfate and physical electrophoresis, as well as with high-resolution imaging of Expansion Microscopy with a general microscopy setup (Wassie et al., 2019; Susaki, Takasato, 2021).

Thus, it is quite important to choose the most optimal and effective tissue clearing technique for samples, especially for such complex objects as cerebral organoids.

One of the most convenient and lab-friendly techniques is CLARITY (Clear Lipid-exchanged Acrylamide-hybridized Rigid Imaging/Immunostaining/In situ hybridization-compatible Tissue-hYdrogel). CLARITY was developed in 2013 for obtaining high-resolution information from complex 3D structures, such as the whole mouse brain (Chung, Deisseroth, 2013). Application of this technique enabled to obtain intact-tissue imaging of long-range projections, local circuit wiring, cellular relationships, subcellular structures, protein complexes, and neurotransmitters. CLARITY protocol includes replacing lipids with hydrophilic polymers (acrylamide and bis-acrylamide), which help to stabilize tissue but make it optically transparent and permeable. It is very important that molecules like nucleic acids and proteins stuck in the hydrogel keep their structures and locations. Thus, CLARITY allows combining tissue clearing techniques with immunostaining and in situ hybridization and explores the internal structure of large three-dimensional objects without damaging their integrity. There is only one article in which CLARITY technique was used for cerebral organoid clarifying (Sakaguchi et al., 2019), but without a detailed description. Thus, the aim of our work was optimization of CLARITY protocol in application to cerebral organoids and detailed description of samples preparation for Light-Sheet microscopy.

## Materials and methods

Reagents

1. Acrylamide (PanReac AppliChem, catalogue number: A1090). 2. Agarose D1, low EEO (Life science products, catalogue number: 1932.0025). 3. Bisacrylamide (PanReac AppliChem, catalogue number: A3636). 4. Boric acid (PanReac AppliChem, catalogue number: A2940). 5. ddH2O. 6. Glue (Henkel, catalogue number: 2340344). 7. Parafilm M (Pechiney Plastic Packaging Company, catalogue number: PM 996). 8. Paraformaldehyde (PFA) (Sigma Aldrich, catalogue number: 158127). 9. Phosphate buffer saline (PBS) (VWR Life Science AMRESCO, catalogue number: Am-E404-100). 10. Sodium azide (Sigma Aldrich, catalogue number: S8032). 11. Sodium dodecyl sulfate (PanReac AppliChem, catalogue number: A1112). 12. Triton X-100 (VWR Life Science AMRESCO, catalogue number: Am-O694-0.1). 13. VA044 (Wako, catalogue number: 011-19365). 14. Serological pipets 5, 10, 25 ml (Corning, catalogue number: 4050, 4100, 4250). 15. 1-ml syringe (B. Braun, catalogue number: 9161635S). 16. 2 ml tube (Eppendorf, catalogue number: 0030120094). 17. 5 ml tube (Axygen, catalogue number: SCT-5ML-S). 18. Glass bottle 100 and 500 ml (Rasotherm, catalogue number: 95206001 and 95206003). 19. Syringe filter, 0.22 μm (TTP, catalogue number: 99722). 20. 4′,6-diamidino-2-phenylindole (DAPI) (Sigma Aldrich, catalogue number: D-9542). 21. Antibodies (Table 1).

**Table 1.**
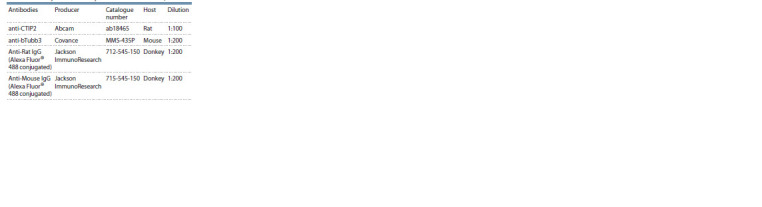
Primary and secondary antibodies used in the protocol

Equipment

1. Light-Sheet Z1 microscope (Zeiss). 2. Orbital shaker (Biosan, catalogue number: OS-20). 3. Roller shaker (Selecta, catalogue number: 7001723). 4. pH meter (OHAUS, catalogue number: 00000032755). 5. Magnetic stirrer (Biosan, catalogue number: MSH-300i). 6. Standard microwave. 7. Thermometer. 8. Forceps. 9. Chemical spoons. 10. Fume hood. 11. Icebox.

Software

1. ImageJ (NIH, https://imagej.nih.gov/ij/index.html) 2. ZEN (Zeiss, https://www.zeiss.com/)

Procedure

Fixation of human cerebral organoid

Note: For any manipulation with organoids, use cut 1 ml tips or wide orifice 1 ml tips to protect samples from damage. 1. Transfer cerebral organoids in 5-ml tubes and wash with 1X PBS solution 2 times. 2. Replace 1X PBS solution with freshly prepared 4 % PFA solution. 3. Place the tubes on the roller/orbital shaker and incubate at room temperature for 2–3 h. 4. Wash samples 3 times with 1X PBS solution for 30 min. Note: At this step, cerebral organoids can be kept at +4 °C in 1X PBS solution. For keeping more than 1 week, we recommend adding sodium azide to a final concentration of 0.01 % to prevent sample contamination with bacteria and fungi.

Hydrogel embedding

1. Precool all solutions, equipment, and samples on ice to prevent premature polymerization of the hydrogel solution. Note: If you use a frozen aliquot of hydrogel solutions, thaw the vial on ice in a fridge overnight. After thawing, gently mix and check for the absence of precipitation. 2. Fill the 2-ml tube with the hydrogel solution and transfer cerebral organoids in the tube having previously gently removed leftovers of the PBS with a paper towel. Note: 2-ml tube format is acceptable for 1–3 organoids. For a large number of organoids, we recommend using a bigger tube. 3. Incubate the samples in the hydrogel solution at +4 °C at the lowest speed of roller/orbital shaker for 24 h. 4. Refill the tube with fresh hydrogel solution and incubate at +37 °C for 4 h. Note: Fill the tube with hydrogel solution completely. Oxygen inhibits hydrogel polymerization, thus all bubbles should be removed. Additionally, we recommend covering the tube with Parafilm to prevent air access. 5. Very gently extract the samples from the polymerized hydrogel by carefully rolling samples on a paper towel. 6. Transfer the samples into the 5-ml tube and wash with Clearing Solution 4 times at room temperature for 24 h.

Passive clearing

1. Change Clearing Solution every 2 days and incubate at +37 °C with agitation. Continue clearing until samples become transparent. Note: We strongly recommend using +37 °C for lipid removal. Room temperature slows this process down to several months! Note: The time of tissue clearing depends on the size of organoids. Cerebral organoids ≤0.5 cm become transparent during ~2 weeks, for organoids ≥0.5 cm it can take up to 3 weeks. 2. Wash samples in PBST for 48 h. Change solution 2–3 times per day.

Staining

1. Incubate samples with primary antibodies in PBST at room temperature on a shaker for 3 days. 2. Wash samples with PBST for 2 days, changing PBST every 4 h. 3. Incubate with secondary antibodies and DAPI at room temperature for 2 days. 4. Wash samples with PBSR for 2 days, changing PBST every 4 h. Note: Antibody consumption for staining of CLARITY samples is very high. We recommend reducing the volume to the minimum at which the samples in the tube are completely covered with the staining buffer with constant stirring on an orbital or roller shaker. Note: For larger organoids, we recommend extending each staining step by at least 1 day

Sample preparation for Light-Sheet microscopy

**Fig. 1.**
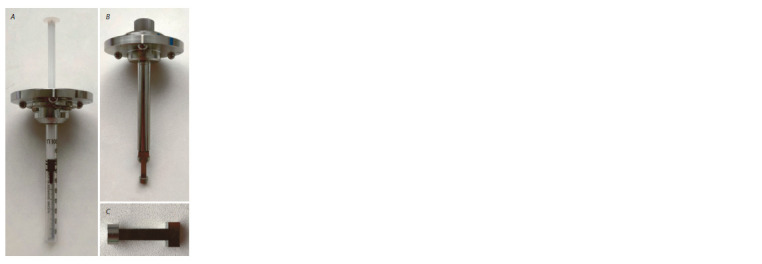
A, ready-to-use sample holder with 1-ml syringe for agarose embedded samples; B, sample holder for hanging samples; C, the bar for sample sticking.

Organoid sizes can vary greatly. Therefore, we recommend using a different fixation method for Light-Sheet microscopy depending on the size. Agarose embedding samples (for smaller samples) Note: Use the agarose with a low melting point temperature only. Note: The percentage of agarose solutions depends on the size of the organoid. For larger organoids, use 1.5 % agarose solution. 1. Prepare the 1-ml syringe by cutting off the top (Fig. 1, A). 2. Weigh the required amount of agarose (at the rate of 1 g per 100 ml) and dissolve in 1X PBS or ddH2O. Prepare agarose solution by melting in the microwave. Usually, for a 1-ml syringe, 1.5 ml of agarose is enough. 3. Pour the hot agarose solution into a 12-well plate or any other laboratory glassware or plasticware. When agarose solution cools down to +40 °C, transfer samples and gently mix. Put the samples in agarose solution into the 1-ml syringe. 4. Assemble the 1-ml syringe with a sample holder (see Fig. 1, A). 5. Proceed to Light-Sheet microscopy. Note: Fill the microscope chamber with ddH2O or 1X PBS. No great differences were observed between the two solutions. Agarose-free or hanging samples (for bigger samples)

1. Glue the sample to the bar (see Fig. 1, B, C). The area of adhesion can be increased by attaching a small piece of filter paper. Keep samples in ddH2O or 1X PBS before placing them into the microscope chamber. 2. Proceed to Light-Sheet microscopy. Note: It is imperative to check and rinse the rod and sample holder for glue residues. If there are any, we strongly recommend that you soak in soapy water and mechanically remove any glue residue. Recipes Note: Most solutions and reagents from this protocol are toxic and biohazardous. Do not forget about your safety and work in protective laboratory clothing and only under a fume hood! 10X PBS solution To prepare a 10X stock solution, dissolve 10 tablets of PBS in 100 ml of ddH2O. PFA solutions • 16 % PFA stock solution To prepare stock solution, dissolve 16 g of PFA in 80 ml of 1X PBS using a magnetic stirrer. Adjust pH to 7.4–7.5 and add 1X PBS up to 100 ml. Filter the solution through a 0.40 μm filter and aliquote into 5 ml tubes. Keep stock solution at +4 °C for short storage (up to 2 weeks) or at –20 °C for long storage. • 4 % PFA working solution To prepare 4 % PFA working solution, dilute stock solution with 1X PBS. Hydrogel solution Note: All solutions and equipment have to be pre-cooled to prevent premature polymerization of hydrogel solution. 1. Mix all components on ice according to Table 2. 2. Aliquote hydrogel solution and keep at –20 °C for long storage or use freshly prepared solution.

**Table 2:**
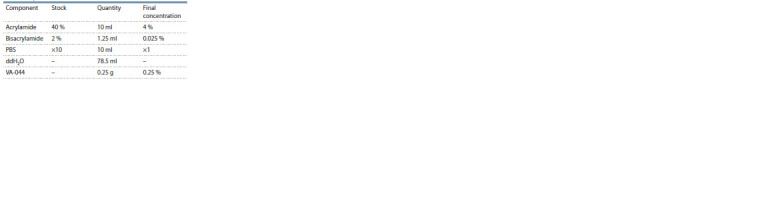
Hydrogel solution composition

Clearing solution 1. Mix all components on ice according to Table 3. 2. Keep the solution in a glass bottle at room temperature.

**Table 3:**
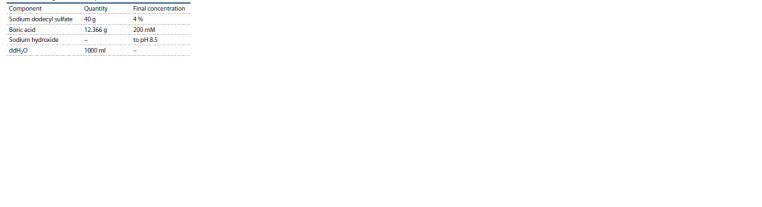
Clearing solution composition

PBST 1. Add Triton-X100 to the final concentration of 0.1 % using a magnetic stirrer. 2. Keep the solution in a glass bottle at room temperature.

## Results

**Fig. 2.**
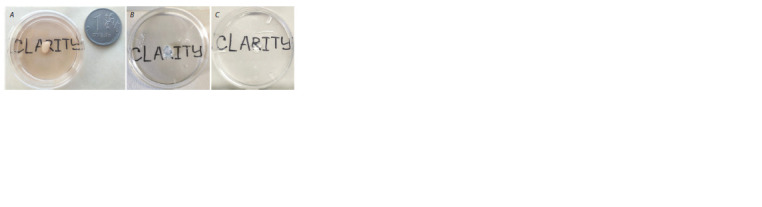
A, intact cerebral organoid before CLARITY; В, hydrogel embedded cerebral organoid before tissue clearing; C, cerebral organoids after 2 weeks of tissue clearing.

Human cerebral organoids were generated according to a protocol from Lancaster et al. (2013) with small modifications. 2- and 3-month-old cerebral organoids were used for tissue clearing protocol. At this stage, there are dense spheres more than 2 mm in diameter (Fig. 2, A). We noted that the time of tissue clearing depends on the size of organoids. Cerebral organoids ≤ 0.5 cm become transparent during ~2 weeks, for organoids ≥ 0.5 cm up to 3 weeks. This time may vary from sample to sample, however, continue cleaning until the samples become transparent (see Fig. 2, B, C).

**Fig. 3.**
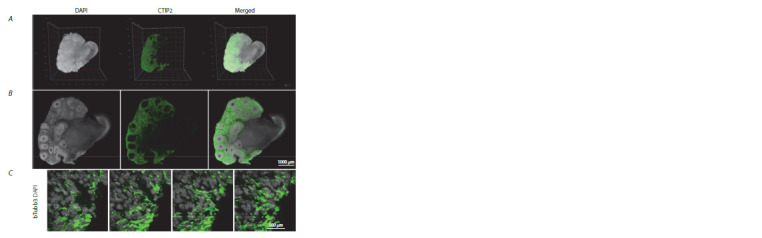
Light-Sheet imaging of a cerebral organoid after CLARITY: A, 3D reconstructed cerebral organoid, 5×, NA 0.16, water immersion; B, optical section of the middle part of the cerebral organoid, 5×, NA 0.16, water immersion; C, optical sections of a small part of the cerebral organoid, 10×, NA 0.5, water immersion.

For immunostaining we chose two proteins with different subcellular localisation such as nuclear CTIP2 (Fig. 3, A) and cytoplasmic bTubb3 (see Fig. 3, C). We did not find a significant difference between penetration of antibodies into different cellular compartments. In both cases, we observed specific staining throughout the entire thickness of the organoid (see Fig. 3, B).

## Conclusion

Cerebral organoids are a unique novel technology that allows the reconstruction of early human neurogenesis. The outstanding feature of this in vitro system is the reproduction of the three-dimensional organization of the human embryonic brain. Standard histological methods of analysis do not allow reconstructing the internal structure of the cerebral organoids and result in information loss. The tissue clearing technique helps to overcome these limitations, allowing to recreate a three-dimensional model of cerebral organoids and explore their fine organization without internal structure destruction. This is especially important for the investigation of brain organoids since they contain a dense network of long processes of nerve cells, which is very difficult to study by serial sections (Dodt et al., 2007).

Based on the various tissue clearance techniques analysis, we settled on the use of hydrogel-tissue chemistry as a clearing agent. Generally hydrophobic and hydrophilic reagent-based protocols are applied to the investigation of spheroids or hollow organoids such as intestinal organoids (Susaki, Takasato, 2021), while hydrogel reagents are used for the clarifying of human iPSC-derived retinal organoids (Cora et al., 2019) and iPSC-derived cerebral organoids (Renner M. et al., 2017; Sakaguchi et al., 2019; Albanese et al., 2020). Hydrogel-tissue chemistry-based protocols maximize the preservation of the internal structure of organoids and allow to achieve high optical resolution and low background at fluorescent microscopy

Currently, there are at least three known hydrogel-tissue chemistry-based methods that use different delipidation and dehydration chemicals: SWITCH (Glutaraldehyde crosslinking (Delipidation) Diatrizoic acid N-methyl-D-glucamine Iodixanol (dehydration)), SHIELD (Polyepoxy cross-linking (Delipidation), Diatrizoic acid N-methyl-D-glucamine Iodixanol (dehydration)) and CLARITY (Hydrogel embedding (Delipidation), HistodenzTM Glycerol (dehydration)) (Susaki, Takasato, 2021; Yu et al., 2021). Therefore in our choice of a suitable technique, we also focused on the availability of the appropriate reagents, the simplicity of the protocol and the lack of need for special equipment

Of course, a significant disadvantage of CLARITY technique is the relatively long tissue clearance procedure (approximately three weeks for 90-days cerebral organoids), but this obstacle is compensated by a quite simple protocol. To our knowledge, there is a single report in which the CLARITY technique was used for cerebral organoid clarifying (Sakaguchi et al., 2019); however, a detailed description of this technique applied to brain organoids has not been previously performed. For the first time, we make a detailed description of the human cerebral organoid samples preparation for investigation of CLARITY-treated samples for Light-Sheet microscopy.

## Conflict of interest

The authors declare no conflict of interest.
